# Androgen receptor gene amplification and protein expression in hormone refractory prostate cancer

**DOI:** 10.1038/sj.bjc.6601127

**Published:** 2003-07-29

**Authors:** J Edwards, N S Krishna, K M Grigor, J M S Bartlett

**Affiliations:** 1 Endocrine Cancer Group, Surgical and Translational research section, Division of Cancer Sciences and Molecular Pathology, Glasgow Royal Infirmary, Glasgow G31 2ER, Scotland; 2 University Department of Pathology, Edinburgh Royal Infirmary, Edinburgh EH8 9AG, Scotland

**Keywords:** hormone-resistant prostate cancer, androgen receptor, gene amplification

## Abstract

This study examined androgen receptor (AR) gene amplification and protein expression in 102 matched paired hormone sensitive and resistant tumours from 51 patients. AR gene amplification and X chromosome copy number were assessed by fluorescent *in situ* hybridisation, and protein expression was assessed by immunohistochemistry. All tumours were stained for PSA protein expression. Significantly more tumours exhibited AR amplification following the development of hormone resistance (20%, 10 out of 49) compared to matched hormone-sensitive tumours from the same patient (2%, one out of 48) (*P*=0.0085). The level of AR expression was significantly higher in hormone-resistant tumours compared to matched hormone-sensitive tumours from the same patient (130, interquartile range, 55–167 *vs* 94.5 interquartile range, 55–120, *P*=0.019). AR expression levels in hormone-resistant tumours with and without AR amplification were not significantly different. However, an increase in AR expression was seen with the development of AR amplification in paired tumours. The rate of AR gene amplification and/or an increase in AR protein expression during androgen resistant is too low to wholly explain the development of androgen resistance. Alternative mechanisms for modulating the function of the AR, or other signalling pathways, must be considered as key factors in the development of hormone-resistant prostate.

Prostate cancer is the second most frequent cause of male cancer-related deaths (Goktas *et al*, 1999). Androgens regulate prostate gland growth and differentiation by binding to the androgen receptor (AR), which regulates a network of androgen responsive genes for example, PSA. Prostate cancer growth is also stimulated by androgens (Gregory *et al*, 1998), and can be inhibited by AR antagonists (antiandrogens) or surgical castration (Avila *et al*, 2001). Approximately 70–80% of prostate cancer patients treated with antiandrogens respond favourably in the first instance (Goktas *et al*, 1999). This effect is however, transient (Newling *et al*, 1997) with the majority of patients developing androgen resistance (Newling *et al*, 1997). The mechanisms involved with the development of resistance are poorly understood, but AR mutations (Avila *et al*, 2001), AR amplification (Visakorpi *et al*, 1995), increased AR expression (Gregory *et al*, 1998) and activation of the AR by interaction with other signalling pathways have been implicated (Culig *et al*, 1998).

Androgen receptors are present in all epithelial cells of the prostate. Downregulation of AR expression during prostate cancer progression (Segawa *et al*, 2001) and increased expression with the development of hormone refractory tumours (Trapman and Cleutjens, 1997; Latil *et al*, 2001; Linja *et al*, 2001) have both been reported. AR expression has been reported to predict which patients will respond to hormone therapy and to correlate with tumour grade, stage and progression-free survival (Trapman and Cleutjens, 1997; Koivisto and Helin, 1999; Linja *et al*, 2001) . It has been postulated that AR protein expression is increased due to AR amplification by a gene dosage effect resulting in the development of androgen resistance. AR gene copy number has been demonstrated to correlate positively with PSA expression (Koivisto and Helin 1999). Polysomy of chromosome X ranges from 42 to 60% and amplification of the AR gene ranges from 20 to 30% in hormone-resistant prostate tumours (Koivisto *et al*, 1997; Bubendorf *et al*, 1999; Miyoshi *et al*, 2000; Edwards *et al*, 2001). However, there is still no clear investigation into AR gene amplification and AR protein expression in hormone-sensitive and hormone-resistant prostate cancer, using paired tumours from the same patient.

## MATERIALS AND METHODS

### Patients

A total of 51 patients (102 matched tumours) were retrospectively selected for analysis; all tumours had patient identification removed including block number and hospital number and were coded in order to make the database anonymous. In 20 cases, AR fluoresence *in situ* hybridisation results only have been previously reported (Edwards *et al*, 2001). Ethical approval was obtained from the multicentre research ethics committee (MREC) for Scotland and the appropriate local research and ethical committees (LREC) for use of matched hormone-sensitive and hormone-resistant tumours in our study. All patients received conventional androgen deprivation therapy (orchidectomy, antiandrogens or androgen ablation therapy). Patients were selected for analysis if they initially responded to treatment (response was defined by prostate-specific antigen (PSA) levels falling by at least 50%), but subsequently relapsed. Patients were classed as having hormone escaped cancer when sustained rising PSA levels were noted and were selected for study if a post-hormone relapse sample was available. The initial tumour sample was either a transurethral resection of the prostate (TURP) or a transrectal ultrasound-guided biopsy (TRUS); however, the relapsed tumour sample was always a TURP which was performed to treat clinical symptoms. PSA values and full clinical follow-up was available for each patient, tumours were assigned Gleason Scores by a single pathologist (KMG).

### Fluorescence *in situ* hybridisation

Sections (5 *μ*m) cut from archival formalin-fixed, paraffin-embedded tissue were placed on aminopropyltriethoxysilane (silane)-treated slides. The slides were pretreated on a VP2000 robotic slide processor (Vysis, UK, Ltd), as previously described^14^ using dual labelling (X chromosome, Spectrum Green™ labelled CEP X, Vysis, UK, Ltd and AR, Spectrum Orange™ labeled probe locus Xq11-13, Vysis, UK, Ltd).

The normal range for X chromosome and AR copy number were identified using the mean chromosomal copy number from 14 benign prostatic hyperplasia (BPH). Amplification was defined as an AR : X ratio greater than 1.5 (Edwards *et al*, 2001).

### Immunohistochemistry (IHC)

Prostate-specific antigen and AR protein expression was determined in paraffin-embedded tissue sections by IHC with a standard immunoperoxidase procedure. Antigen retrial was performed by microwaving under pressure for 5 min in TE buffer. A purified immunoglobulin fraction of either a rabbit antiserum to human PSA (A 0562, DAKO, Denmark 2 *μ*g ml^−1^) or AR (NCL-AR-2F12, Vector, UK, 1 *μ*g ml^−1^) was used as the primary antibodies. In each case, an isotype-matched antibody was used as a negative control in which no staining was observed. Positive control sections were included in each IHC assay. Bound antibody was visualised by the streptavidin–biotin (ABC kit, Vector Labs, UK) method including diaminobenzidine as a chromogen (DAKO).

Staining was scored blind by two independent observers using a weighted histoscore method (Fraser *et al*, 2003), interclass correlation coefficients (ICCC) were calculated and confirmed acceptable correlation between observer scores (Armitage and Berry, 1995). ICCC values for histoscores were 0.67 (good) and 0.91 (excellent) for PSA and AR staining, respectively (Armitage and Berry, 1995). Staining was defined as increased or decreased only in those samples with a difference between paired samples of greater than 2 × the mean observer variation for the parameter under investigation.

### Statistics

The Fisher's exact test was used to compare FISH results pre- and post-hormone resistance. Prostate-specific antigen protein expression and AR protein expression are shown as median and interquartile ranges. Paired Student's *t*-tests were used to compare PSA and AR expression between pre- and post-hormone-resistant tumours. Wilcoxon signed-rank test was used to compare Gleason sum between paired hormone-sensitive and hormone-resistant tumours. Comparisons between the resistant tumours with and without AR gene amplification were made with the Mann–Whitney test and *χ*^2^ test for linear trends. Correlations between AR gene copy number and PSA MHS (mean histoscore) and AR MHS or PSA MHS and AR MHS and Gleason sum were calculated using the Spearman rank test.

## RESULTS

### Patient information

Of the 51 patients analysed, seven had bone metastases at the time of initial hormone therapy: 102 tumours were analysed. Gleason sum in hormone-sensitive tumours was significantly lower than in hormone-resistant tumours (*P*<0.001) (Table 1

**Table 1:** Gleason sum of hormone-sensitive and hormone-resistant prostate tumours

	**Hormone-sensitive prostate cancer**	**Hormone-resistant prostate cancer**	***P*-value**
Gleason sum ⩽6	24% (12/51)	4% (5/51)	
Gleason sum=7	27% (14/51)	12% (7/51)	
Gleason sum ⩾8	49% (25/51)	78% (40/51)	<0.001

*P-*values were obtained comparing Gleason sums between paired hormone-sensitive and hormone-resistant tumours by Wilcoxon signed-ranks test. NB: Some observers question the value of Gleason sums in hormone-relapsed disease.

), although some commentators question the value of Gleason scores in hormone-relapsed disease. Mean patient age at diagnosis was 69 (41–83) years, and median follow-up was 1669 days (interquartile range, 1062–2428). Median time to hormone relapse was 899 (interquartile range, 537–1689).

### FISH results

Figure 1

**Figure 1:**
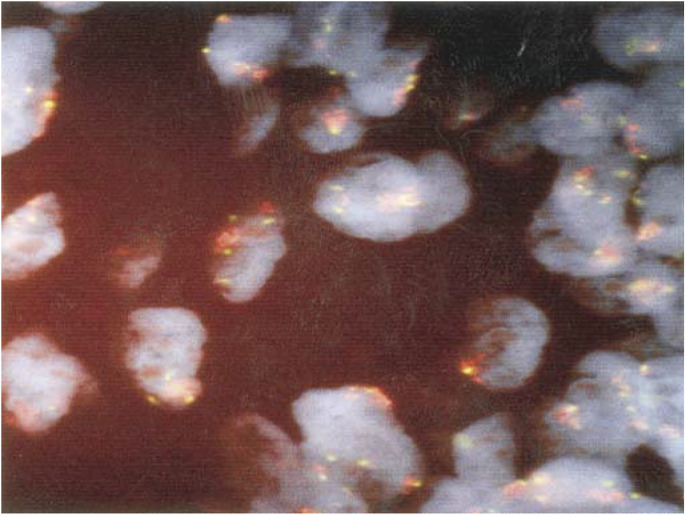
A prostate cancer tumour with AR gene amplification is shown. AR amplification in prostate cancer nuclei (stained with DAPI, blue) showing increased copies of both AR (red) and chromosome X (green). Magnification × 1000.

illustrates an example of a prostate cancer tumour with AR gene amplification. A total of 97 tumours were analysed by FISH (48 hormone-sensitive and 49 hormone-resistant tumours, five cases were not analysed as we were unable to successfully FISH them as they were fixed in Bouins). Significantly more hormone-resistant tumours had AR amplification (20%, 10 out of 49) than hormone-sensitive tumours (2%, one out of 48) (*P*=0.0085) (Table 2

**Table 2:** AR gene amplification rate, AR protein expression and PSA expression in hormone-sensitive and hormone-resistant prostate cancer

	**Hormone-sensitive prostate cancer**	**Hormone-resistant prostate cancer**	***P-*value**
AR copy no.	31% (15/48)	51% (25/49)	0.075
X copy no.	33% (18/48)	47% (23/49)	0.46
Amplified	2% (1/48)	20% (10/49)	0.0085
AR MHS	94.5 (55–120)	130 (55–167)	0.019
PSA MHS	158 (121–185)	132 (101–168)	0.018

AR/X copy no.=percentage of cases with increased AR or chromosome X copy number when compared with BPH. Amplified=percentage of cases with AR : X ratios above 1.5. MHS=median histoscore for androgen receptor or PSA IHC (interquartile ranges in brackets).

). In the amplified tumours, the median AR : X chromosome ratio was 3.11 (interquartile range, 2.4–5.8). In 22 patients, no abnormalities of either the X chromosome or AR copy number were detected in either the biopsy taken before or after hormone relapse. In all, 18 (38%) hormone-sensitive tumours and 23 (47%) hormone-relapsed tumours (*P*=0.46) had increased copies of the X chromosome (Table 2). A total of 15 (31%) hormone-sensitive tumours and 25 (51%) hormone-relapsed tumours (*P*=0.075) showed evidence of increased AR gene copies (Table 2). There was no significant difference between the level of gene or chromosome copy numbers with the development of hormone refractory disease. Gleason sum was not significantly different in hormone-resistant tumours with and without AR amplifications (Table 3

**Table 3:** Gleason sums in hormone-resistant prostate cancer tumours with and without AR amplification

	**AR normal (38 cases)**	**AR amplified (10 cases)**	***P*-value**
Gleason sum ⩽6	23% (9/38)	0% (0/10)	
Gleason sum=7	32% (12/38)	10% (1/10)	
Gleason sum ⩾8	45% (17/38)	90% (9/10)	0.423

AR amplification=AR : X ratio greater than 1.5, AR normal=AR : X ratio within normal range. The Mann–Whitney test was used to establish if there was a significant difference in Gleason sum in tumours with or without AR amplification.

). There was no significant difference in time to relapse in patients with AR amplifications (median 1143, interquartile range, 230–1731) compared to those without AR amplifications (median 857, interquartile range 541–1709).

### IHC results

Figure 2

**Figure 2:**
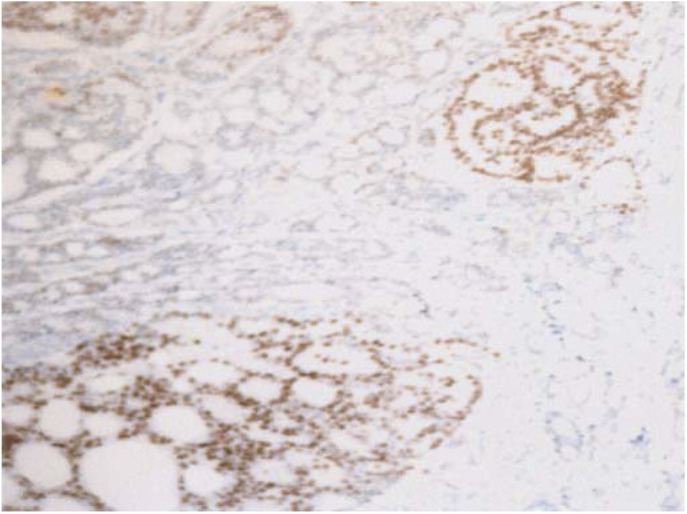
A prostate cancer tumour that expresses AR protein is shown. AR protein expression is coloured brown and is present in the tumour cell nuclei. Magnification × 400.

illustrates an example of AR protein expression in hormone-resistant prostate tumours. The median AR histoscore was 108 (interquartile range, 55–153), the level of AR expression was significantly higher in hormone-resistant tumours compared to matched hormone-sensitive tumours (median 130, interquartile range, 55–167 *vs* median 94.5 interquartile range, 55–120, *P*=0.019) (Table 2) with overlap between groups. There was no significant difference in time to relapse in patients where AR expression increased (median 907, interquartile range 485–1653) compared to those where AR expression was unchanged or decreased (median 836, interquartile range, 496–1914).

Four samples did not express AR protein. In one of the hormone-resistant cases, the previous hormone-sensitive biopsy showed both AR amplification (2.9) and a mean AR histoscore of 140. Following the development of androgen resistance, the AR amplification ratio did not change (3.09); however, no AR expression was detected in either of the two post-treatment biopsies from this patient. PSA expression remained unchanged in the transition from hormone-sensitive to hormone-resistant disease (Table 2).

AR expression levels in hormone-resistant tumours with and without AR amplification are not significantly different (Table 4

**Table 4:** Comparison of AR and PSA expression levels in hormone-resistant prostate cancer with and without AR amplification

	**AR normal (38 cases)**	**AR amplified (10 cases)**	***P*-value**
Median age (years)	70 (67–75)	66 (63–70)	0.29
Time to relapse	857 (541–1709)	1641 (306–1746)	0.6
AR IHC	121 (53–156)	172 (129–191)	0.9
PSA IHC	135 (109–160)	97 (85–162)	0.3

**Figure 3:**
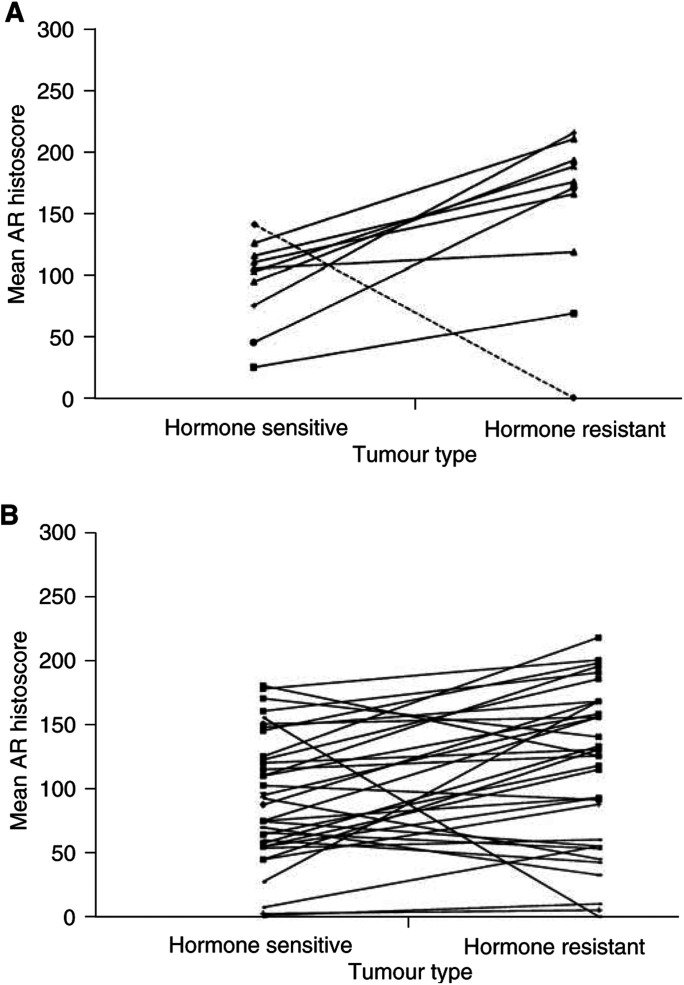
(**A**) The AR protein mean histoscore for matched hormone-sensitive and hormone-resistant tumours with AR amplification is shown. The cases shown with unbroken lines are those cases that developed AR amplification in the transition from hormone-sensitive to hormone-resistant disease. All cases had an increase in AR protein expression with the development of AR amplification, However, only eight out of the nine cases significantly increased. The case shown with the broken line had AR gene amplification in the hormone-sensitive and hormone-resistant tumour. This case had a significant decrease in AR protein expression with the development of hormone resistance. (**B**) The AR protein mean histoscore for 41 matched hormone-sensitive and hormone-resistant tumours with out AR amplification are shown. In 14 out of 41 (35%) cases, AR expression increased markedly with the development of hormone relapse.

AR amplified=AR : X ratio greater than 1.5, AR normal=AR : X ratio within normal range; median age=median age at diagnosis of AR amplified or normal cases (interquartile range in brackets); time to relapse=median time to relapse (in days) of AR amplified or normal cases (interquartile range in brackets); AR/PSA IHC=median AR or PSA histoscores for both groups (interquartile ranges in brackets).

). However, an increase in AR expression was seen in 80% (eight out of 10) of the cases (Figure 3A

) with AR amplification compared to only 35% of cases without AR amplification (Figure 3B). Using Spearman's rank correlation, AR expression in hormone-sensitive and hormone-resistant prostate cancer did not correlate with either Gleason sum or PSA expression.

All tumours included in this study expressed PSA. The median PSA histoscore was 146 (range=108–167), the level of PSA expression was significantly lower in hormone-resistant tumours compared to matched hormone-sensitive tumours (*P*=0.018; Table 2).

## DISCUSSION

Androgen receptor gene amplifications are uncommon in hormone-sensitive tumours and are present in 20–30% of hormone-resistant tumours (Visakorpi *et al*, 1995; Koivisto *et al*, 1997; Bubendorf *et al*, 1999; Miyoshi *et al*, 2000; Edwards *et al*, 2001). In the present study, we investigated AR amplification, AR protein expression and PSA expression in paired hormone-sensitive and hormone-resistant tumours from the same patient with documented initial responses to androgen deprivation therapy. We confirm here that a significant increase in AR gene amplification rates is seen in the transition from hormone-sensitive to hormone-resistant disease (*P*=0.0085); however, this is only observed in a proportion of cases. Based on our results and those from previous studies between 70 and 80% of cases with hormone relapse must therefore involve mechanisms other than amplification of the AR. While patients with AR gene amplification may represent a readily identifiable, and possibly treatable, group more research should be focused on alternative mechanisms of hormone escape. This is especially relevant as we have also identified a patient with AR amplification in the hormone-sensitive tumour, which responds fully to therapy, suggesting that AR amplification does not preclude a response to androgen deprivation therapy. In the light of this observation it is possible, though at present unproven and to our view unlikely, that AR amplification does not mediate hormone resistance.

It is hypothesised that AR gene amplification is involved in the development of hormone-resistant prostate cancer due to amplification, resulting in an increase in AR protein expression (Visakorpi *et al*, 1995). Real-time RT–PCR demonstrated that even one additional copy of the AR gene may increase AR expression (Linja *et al*, 2001), suggesting that even a small increase in relative gene dosage could have biological significance. However, in the study by Linja no correlation between AR mRNA and protein levels was performed. Similarly in a separate study, Latil *et al*, (2001) also describe increased AR mRNA levels in hormone escaped tumours. Both studies are important in that they demonstrate increases in AR mRNA in prostate cancers. However, neither study documents protein expression or makes use of paired samples from individual patients. An increase in AR protein expression is postulated to enable low circulating levels of androgens that are present following orchidectomy or treatment with LHRH agonists, to activate the AR even in the presence of antiandrogens (Koivisto *et al*, 1997). We therefore measured AR protein expression as well as AR amplification status in our patient cohort. As we have a unique data set of matched hormone-sensitive and hormone-resistant tumours from each patient and therefore enabling us to follow AR protein expression with the development of resistance in the same patient and relate this to AR amplification status, stringent quality controls (see above) were included to validate IHC results. Our study confirms that AR expression increases as the disease progresses to its hormone refractory state (*P*=0.019) and that 80% of cases in which AR amplification was observed also exhibited an increase in AR expression. However, an increase in AR expression was also seen in 35% of cases that did not develop AR amplification. This suggests that although an increase in AR expression is associated with AR amplification, an increase in expression may also be due to alternative mechanisms, for example, decrease in protein degradation or an increase in protein stabilisation. Almost half of all patients do not show any increase in AR expression during the development of hormone-resistant disease. We also identified a patient with AR amplification in both hormone-sensitive and hormone-resistant tumours and three cases with loss of AR protein expression at the development of hormone resistance, as observed by Gnanapragasam *et al*, (2002). Therefore, increased androgen receptor expression, by whatever mechanism, can at best only explain hormone resistance in a subgroup of prostate cancer cases. Alternative mechanisms of hormone resistance are activation of AR via phosphorylation (Zhu and Liu 1997; Lin *et al*, 2001), therefore increasing AR activity without effecting its expression levels or may bypass the AR, for example, AP-1 phosphorylation (Henttu and Vihko, 1998; Krishna *et al*, 2002).

While PSA expression was significantly lower in hormone-sensitive tumours compared to hormone-resistant tumours (*P*=0.018), this decrease was marginal with considerable overlap between values for pre- and post-hormone-resistant tumours (Table 1). The significance of this finding is therefore unclear and requires further investigation.

This is the first study to our knowledge that has correlated AR gene amplification, AR protein expression and PSA protein expression in matched hormone-sensitive and hormone-resistant prostate cancer. We have confirmed that AR gene amplification is associated with the development of hormone resistance in 20% of patients and this is related to an increase in AR expression in the majority of cases. We have also demonstrated that this is not the only mechanism by which AR expression is increased and also that in almost 60% of cases, AR expression remains unchanged or decreases. Therefore, in these cases, hormone resistance must be due to alternative mechanisms of AR activation or may be due to mechanisms that can stimulate AR responsive genes independent of the AR.
